# Influenza Activity — United States, 2014–15 Season and Composition of the 2015–16 Influenza Vaccine

**Published:** 2015-06-05

**Authors:** Grace D. Appiah, Lenee Blanton, Tiffany D’Mello, Krista Kniss, Sophie Smith, Desiree Mustaquim, Craig Steffens, Rosaline Dhara, Jessica Cohen, Sandra S. Chaves, Joseph Bresee, Teresa Wallis, Xiyan Xu, Anwar Isa Abd Elal, Larisa Gubareva, David E. Wentworth, Jacqueline Katz, Daniel Jernigan, Lynnette Brammer

**Affiliations:** 1 Influenza Division, National Center for Immunization and Respiratory Diseases, CDC

During the 2014–15 influenza season in the United States, influenza activity* increased through late November and December before peaking in late December. Influenza A (H3N2) viruses predominated, and the prevalence of influenza B viruses increased late in the season. This influenza season, similar to previous influenza A (H3N2)–predominant seasons, was moderately severe with overall high levels of outpatient illness and influenza-associated hospitalization, especially for adults aged ≥65 years. The majority of circulating influenza A (H3N2) viruses were different from the influenza A (H3N2) component of the 2014–15 Northern Hemisphere seasonal vaccines, and the predominance of these drifted viruses resulted in reduced vaccine effectiveness (1). This report summarizes influenza activity in the United States during the 2014–15 influenza season (September 28, 2014–May 23, 2015)† and reports the recommendations for the components of the 2015–16 Northern Hemisphere influenza vaccine.

## Viral Surveillance

**FIGURE 1:**
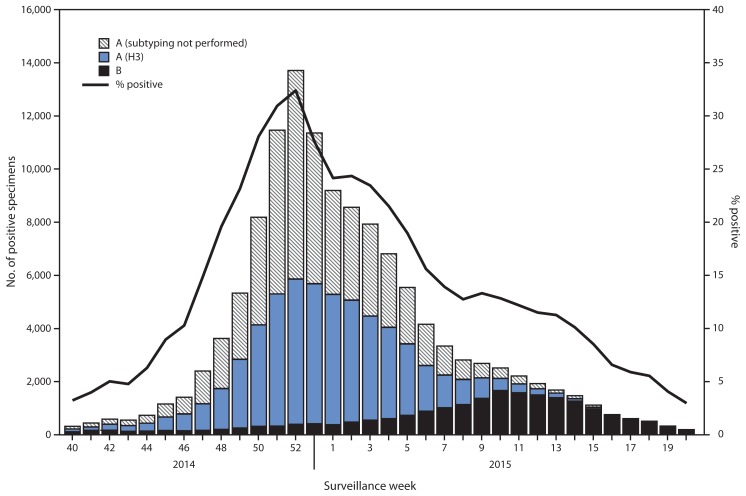
Number* and percentage of respiratory specimens testing positive for influenza reported by World Health Organization and National Respiratory and Enteric Virus Surveillance System collaborating laboratories, by type, subtype, and surveillance week — United States, 2014–15 influenza season^†^ * N = 125,462. ^†^ Data as of May 23, 2015.

During September 28, 2014–May 23, 2015, World Health Organization (WHO) and National Respiratory and Enteric Virus Surveillance System collaborating laboratories in the United States tested 691,952 specimens for influenza viruses; 125,462 (18.1%) were positive (Figure 1). Of the positive specimens, 104,822 (83.5%) were influenza A viruses, and 20,640 (16.5%) were influenza B viruses. Among the seasonal influenza A viruses, 52,518 (50.1%) were subtyped; 52,299 (99.6%) were influenza A (H3N2) viruses, and 219 (0.2%) were A (H1N1)pdm09 viruses. In addition, three variant influenza A viruses§ (one H3N2v and two H1N1v) were identified.

Through the peak of the 2014–15 season, H3N2 viruses predominated nationally, with lesser numbers of influenza B viruses and influenza A (H1N1)pdm09 viruses also identified. Based on the percentage of specimens testing positive for influenza to determine the peak of influenza activity, the peak occurred during week 52 (the week ending December 27, 2014) nationally; however, differences among U.S. Department of Health and Human Services regions¶ were observed in the timing of influenza activity and relative proportions of circulating viruses. Activity in region 7 peaked earliest, during the week ending December 13, 2014 (week 50), and activity in region 1 peaked latest, during the week ending January 24, 2015 (week 3).

Although H3N2 activity peaked between late December and early January, substantial influenza B activity occurred late in the season. Influenza A viruses predominated until late February, with influenza B viruses predominating from the week ending February 28, 2015 (week 8) through the week ending May 23, 2015 (week 20). The highest proportion of influenza B viruses was observed in Region 4 (19.8%), and the lowest proportion of influenza B viruses was detected in Region 10 (11.1%).

## Novel Influenza A Viruses

During the 2014–15 influenza season, three cases of human infection with novel influenza A viruses have been reported. One infection with an influenza A (H3N2) variant virus occurred during the week ending October 18, 2014 (week 42) in Wisconsin, and one infection with an influenza A (H1N1) variant (H1N1v) virus was reported to CDC during the week ending January 24, 2015 (week 3) from Minnesota. Both patients had illness onset in October 2014 and reported contact with swine in the week preceding illness. Both patients fully recovered, and no further cases were identified in contacts of either patient. The third case, a fatal infection with an H1N1v virus was reported from Ohio during the week ending April 2, 2015 (week 17). The patient worked at a livestock facility that housed swine, but no direct contact with swine in the week before illness onset was reported. The patient died from complications of the infection, and no ongoing human-to-human transmission was identified.

## Antigenic and Genetic Characterization of Influenza Viruses

WHO collaborating laboratories in the United States are requested to submit a subset of their influenza-positive respiratory specimens to CDC for further virus characterization. CDC has antigenically and/or genetically characterized** 2,193 influenza viruses collected and submitted by U.S. laboratories since October 1, 2014, including 59 influenza A (H1N1)pdm09 viruses, 1,324 influenza A (H3N2) viruses, and 810 influenza B viruses. Of the 59 influenza A (H1N1)pdm09 viruses tested, all were antigenically similar to A/California/7/2009, the influenza A (H1N1) component of the 2014–15 Northern Hemisphere influenza vaccine.

A total of 246 (18.6%) of the 1,324 H3N2 viruses tested have been characterized as A/Texas/50/2012-like, the influenza A (H3N2) component of the 2014–15 Northern Hemisphere influenza vaccine. A total of 1,078 (81.4%) of the 1,324 viruses tested showed either reduced titers with antiserum produced against A/Texas/50/2012 or belonged to a genetic group that typically shows reduced titers to A/Texas/50/2012. The viruses that showed reduced titers to A/Texas/50/2012 belonged to multiple genetic groups; most but not all were antigenically similar to the influenza A (H3N2) virus selected in September 2014 for the 2015 Southern Hemisphere and in February 2015 for the 2015–16 Northern Hemisphere influenza vaccines, A/Switzerland/9715293/2013. A total of 948 of the 1,324 A (H3N2) viruses were further characterized; 889 (93.7%) were antigenically similar to A/Switzerland/9715293/2013, and fifty-nine (6.2%) showed reduced titers with antiserum produced against A/Switzerland/9715293/2013 virus.

Of the 810 influenza B viruses tested, 582 (71.9%) belonged to the B/Yamagata lineage, and the remaining 228 (28.1%) influenza B viruses tested belonged to the B/Victoria/02/87 lineage. A total of 571 (98.1%) of the 582 B/Yamagata-lineage viruses were characterized as B/Massachusetts/2/2012-like, which was included as an influenza B component of the 2014–15 Northern Hemisphere trivalent and quadrivalent influenza vaccines. Eleven (1.9%) of the B/Yamagata-lineage viruses tested showed reduced titers to B/Massachusetts/2/2012. Among the 582 B/Yamagata lineage viruses characterized, 576 (98.9%) viruses were antigenically similar to B/Phuket/3073/2013 virus, the B/Yamagata lineage virus selected for the 2015 Southern Hemisphere influenza vaccine and 2015–16 Northern Hemisphere influenza vaccine. Six (1.0%) showed reduced titers with antiserum produced against B/Phuket/3073/2013 virus. A total of 223 (97.8%) of the 228 B/Victoria-lineage viruses were characterized as B/Brisbane/60/2008-like, the virus that is included as an influenza B component of the 2014–15 Northern Hemisphere quadrivalent influenza vaccine. Five (2.2%) of the B/Victoria-lineage viruses tested showed reduced titers to B/Brisbane/60/2008.

## Antiviral Resistance to Influenza Viruses

Since October 1, 2014, a total of 4,192 influenza virus specimens have been tested for resistance to influenza antiviral medications. All 896 influenza B viruses and 3,232 influenza A (H3N2) viruses tested were sensitive to oseltamivir and zanamivir. All 896 influenza B viruses and 1,723 influenza A (H3N2) viruses tested were sensitive to peramivir. Among 64 pH1N1 viruses tested for resistance, one (1.6%) was found to be resistant to oseltamivir and one (1.6%) to peramivir. All 58 influenza A (H1N1)pdm09 viruses tested for resistance to zanamivir were sensitive. High levels of resistance to the adamantanes (amantadine and rimantadine) persist among influenza A viruses currently circulating globally (the adamantanes are not effective against influenza B viruses).

## Composition of the 2015–16 Influenza Vaccine

The Food and Drug Administration’s Vaccines and Related Biological Products Advisory Committee has recommended that the 2015–16 influenza trivalent vaccines used in the United States contain an A/California/7/2009 (H1N1)pdm09-like virus, an A/Switzerland/9715293/2013 (H3N2)-like virus, and a B/Phuket/3073/2013-like (B/Yamagata lineage) virus. It is recommended that quadrivalent vaccines, which have two influenza B viruses, contain the viruses recommended for the trivalent vaccines, as well as a B/Brisbane/60/2008-like (B/Victoria lineage) virus (2). This represents a change in the influenza A (H3) and influenza B (Yamagata lineage) components compared with the composition of the 2014–15 influenza vaccine. These vaccine recommendations were based on several factors, including global influenza virologic and epidemiologic surveillance, genetic characterization, antigenic characterization, antiviral resistance, and the candidate vaccine viruses that are available for production.

## Outpatient Illness Surveillance

**FIGURE 2:**
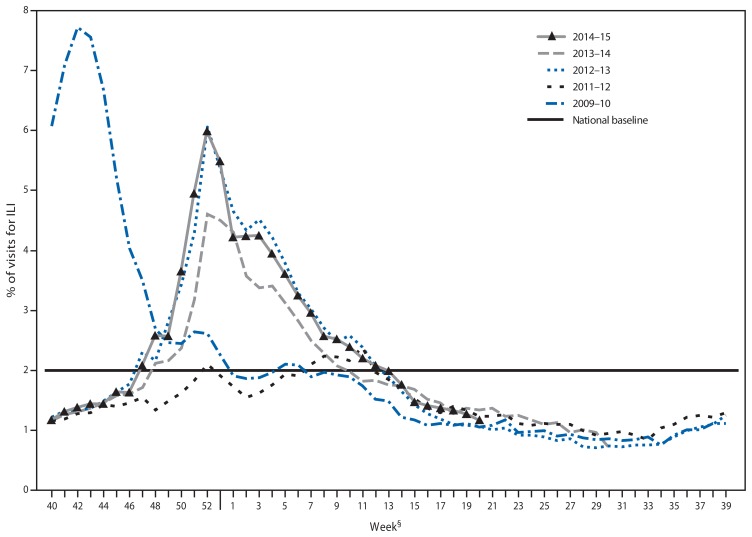
Percentage of visits for influenza-like illness (ILI)* reported to CDC, by surveillance week — Outpatient Influenza-Like Illness Surveillance Network, United States, 2014–15 influenza season and selected previous influenza seasons^†^ * Defined as a fever (≥100.0°F [≥37.8°C]), oral or equivalent, and cough and/or sore throat, without a known cause other than influenza. ^†^ Data as of May 23, 2015. ^§^ Because there was no week 53 in the previous influenza seasons displayed, the week 53 data point for those seasons is an average of percentages from weeks 52 and 1.

Nationally, the weekly percentage of outpatient visits for influenza-like illness (ILI)†† to health care providers participating in the U.S. Outpatient Influenza-Like Illness Surveillance Network (ILINet) was at or above the national baseline level§§ of 2.0% for 20 consecutive weeks during the 2014–15 influenza season (Figure 2). The peak percentage of outpatient visits for ILI was 6.0% and occurred in the week ending December 27, 2014 (week 52). During the 2001–02 through 2013–14 seasons, peak weekly percentages of outpatient visits for ILI ranged from 2.4% to 7.7% and remained at or above baseline levels for an average of 13 weeks (range = 1–19 weeks).

ILINet data are used to produce a weekly jurisdiction-level measure of ILI activity¶¶ ranging from minimal to high. The number of jurisdictions experiencing elevated ILI activity peaked during the weeks ending December 27, 2014 (week 52) and January 24, 2015 (week 3), when a total of 31 states and Puerto Rico experienced high ILI activity. A total of 45 jurisdictions experienced high ILI activity during at least 1 week this season. The peak number of jurisdictions experiencing high ILI activity in a single week during the last five influenza seasons has ranged from four during the 2011–12 season to 44 during the 2009–10 season.

## Geographic Spread of Influenza Activity

State and territorial epidemiologists report the geographic distribution of influenza in their jurisdictions through a weekly influenza activity code.*** The geographic distribution of influenza activity was most extensive during the weeks ending January 3, 2015 (week 53) and January 10, 2015 (week 1), when a total of 47 jurisdictions reported influenza activity as widespread. During the previous five seasons, the peak number of jurisdictions reporting widespread activity has ranged from 20 in the 2011–12 season to 49 in the 2010–11 season.

## Influenza-Associated Hospitalizations

**FIGURE 3:**
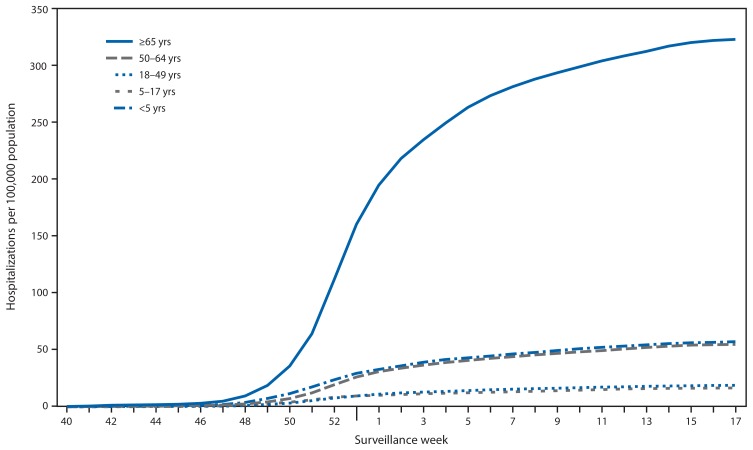
Cumulative rates of hospitalization for laboratory-confirmed influenza, by age group and surveillance week — FluSurv-NET,* United States, 2014–15 influenza season^†^ * FluSurv-NET conducts population-based surveillance for laboratory-confirmed influenza-associated hospitalizations in children aged <18 years (since the 2003–04 influenza season) and adults aged ≥18 years (since the 2005–06 influenza season). FluSurv-NET covers approximately 80 counties in the 10 Emerging Infections Program states (California, Colorado, Connecticut, Georgia, Maryland, Minnesota, New Mexico, New York, Oregon, and Tennessee) and three additional Influenza Hospitalization Surveillance Project states (Michigan, Ohio, and Utah). ^†^ Data as of May 23, 2015.

CDC monitors hospitalizations associated with laboratory-confirmed influenza virus infections using the FluSurv-NET††† surveillance system. Cumulative hospitalization rates (cases per 100,000 population) were calculated by age group based on 17,911 total hospitalizations resulting from influenza during October 1, 2014–April 30, 2015. Among 17,856 cases with influenza type specified, 15,271 (85.5%) were associated with influenza A and 2,473 (13.8%) with influenza B virus and 112 (0.6%) were associated with influenza A and influenza B coinfections; 55 had no virus type information available. Adults aged ≥65 years accounted for approximately 61.0% of reported cases. The cumulative incidence§§§ for all age groups since October 1, 2014, was 65.5 per 100,000 (Figure 3). The cumulative incidence rate (cases per 100,000 population) by age group for this period was 57.2 (0–4 years), 16.5 (5–17 years), 18.9 (18–49 years), 54.8 (50–64 years), and 322.8 (≥65 years). During the past four influenza seasons, age-specific hospitalization rates ranged from 16.0 to 67.0 (0–4 years), 4.0 to 14.6 (5–17 years), 4.2 to 21.5 (18–49 years), 8.1 to 53.7 (50–64 years), and 30.2 to 183.2 (≥65 years).

As of April 30, 2015, among the FluSurv-NET adult patients for whom medical chart data were available, the most frequent underlying conditions were cardiovascular disease (51.0%), metabolic disorders (45.8%) and obesity (33.1%). Among children hospitalized with laboratory-confirmed influenza and for whom medical chart data were available, 43.3% did not have any recorded underlying conditions, and 26.4% had underlying asthma or reactive airway disease. Among the 626 hospitalized women of childbearing age (15–44 years), 200 (31.9%) were pregnant.

## Pneumonia and Influenza-Associated Mortality

**FIGURE 4:**
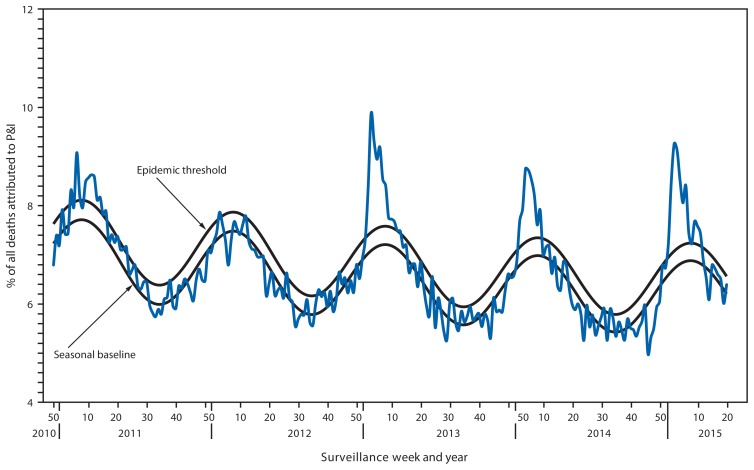
Percentage of all deaths attributable to pneumonia and influenza (P&I), by surveillance week and year* — 122 Cities Mortality Reporting System, United States, 2010–2015 * Data as of May 23, 2015.

During the 2014–15 influenza season, the percentage of deaths attributed to pneumonia and influenza (P&I) exceeded the epidemic threshold¶¶¶ for 8 consecutive weeks from January 3 to February 21, 2015 (weeks 53–7). The weekly percentage of deaths attributed to P&I ranged from 5.0% to 9.3% (Figure 4). The peak weekly percentages of deaths attributed to P&I for the previous five seasons ranged from 7.9% during the 2011–12 season to 9.9% during the 2012–13 season.

## Influenza-Associated Pediatric Mortality

For the 2014–15 influenza season, as of May 23, 2015, a total of 141 laboratory-confirmed, influenza-associated pediatric deaths had been reported from 40 states and New York City. The deaths occurred in 14 children aged <6 months, 23 aged 6–23 months, 22 aged 2–4 years, 45 aged 5–11 years, and 37 aged 12–17 years; mean and median ages were 7.2 years and 5.9 years, respectively. Among the 141 deaths, 109 were associated with an influenza A virus, 29 were associated with an influenza B virus, two were associated with an influenza virus for which the type was not determined, and one was associated with an influenza A and influenza B virus coinfection.

Since influenza-associated pediatric mortality became a nationally notifiable condition in 2004, the total number of influenza-associated pediatric deaths had previously ranged from 34 to 171 per season; this excludes the 2009 pandemic, when 358 pediatric deaths were reported to CDC during April 15, 2009–October 2, 2010.

## Discussion

The 2014–15 influenza season was moderately severe overall and especially severe in adults aged ≥65 years, with predominant circulation of antigenically and genetically drifted influenza A (H3N2) viruses. Influenza activity peaked during late December, with influenza A (H3N2) viruses predominant early in the season through the week ending February 21, 2015 (week 7). Influenza B became the predominant virus starting week 8 (the week ending February 28, 2015). The majority of influenza A (H3N2) viruses sent to CDC for antigenic and/or genetic characterization were different from the influenza A (H3N2) component of the 2014–15 Northern Hemisphere seasonal vaccines (A/Texas/50/2012).

Previous influenza A (H3N2)–predominant seasons have been associated with increased hospitalizations and deaths compared to seasons that were not influenza A (H3N2)–predominant, especially among children aged <5 years and adults aged ≥65 years (3–6). Influenza activity this season was similar to the 2012–13 season, which was the most recent influenza A (H3N2)–predominant season, but with higher rates of influenza-associated hospitalizations among adults aged ≥65 years. The cumulative rate of influenza-associated hospitalizations among this age group was 319.2 per 100,000 population, exceeding the cumulative total of 183.2 per 100,000 population for the 2012–13 season, which had previously been the highest recorded rate of laboratory-confirmed, influenza-associated hospitalizations since this type of surveillance began in 2005. Among children aged <5 years, the cumulative hospitalization rate (57.1 per 100,000 population) was slightly less than that observed during the 2012–13 season (66.2 per 100,000 population). Older adults also accounted for the majority of deaths attributed to P&I this season. Approximately 79.0% of the P&I deaths this season have occurred in adults aged ≥65 years, which is similar to what was observed during the 2012–13 influenza season (79.5%). However, the peak weekly percentage of deaths attributed to P&I for the current influenza season (9.3%) was lower than the peak observed during the 2012–13 influenza season (9.9%).

Influenza vaccination this season offered reduced protection against the predominant circulating viruses, drifted influenza A (H3N2), compared with previous seasons when most circulating and vaccine strain viruses were well-matched. Data collected during November 10, 2014–January 30, 2015, indicated that the influenza vaccine was 19% (95% confidence interval [CI] = 7%–29%) effective in preventing medical visits against all influenza across all age groups, and was 18% (CI = 6%–29%) and 45% (CI = 14%–65%) effective in preventing medical visits associated with influenza A (H3N2) and influenza B (Yamagata lineage), respectively (7). Despite reduced vaccine effectiveness, influenza vaccination was still recommended for all unvaccinated persons aged ≥6 months (8,9). Influenza vaccination provided protection against vaccine-like influenza A (H3N2) viruses that had not undergone significant antigenic drift and against influenza B viruses, which predominated later in the season (1,3,6). Of note, among the influenza A (H3N2) viruses, most, but not all, were antigenically similar to the influenza A (H3N2) virus selected for the 2015 Southern Hemisphere influenza vaccine (A/Switzerland/9715293/2013) (1).

Testing for seasonal influenza viruses and monitoring for novel influenza A virus infections should continue throughout the summer. Although summer influenza activity in the United States is typically low, influenza cases have occurred during the summer months and clinicians should remain vigilant in considering influenza in the differential diagnosis of summer respiratory illnesses. Health care providers also are reminded to consider novel influenza virus infections in persons with ILI, swine or poultry exposure, or with severe acute respiratory infection after travel to areas where avian influenza viruses have been detected. Providers should alert the local public health department if novel influenza virus infection is suspected. Early treatment with influenza antiviral medications is recommended for persons at high risk for influenza-associated complications, as defined by the Advisory Committee on Immunization Practices, or with severe influenza illness. In randomized, controlled trials, antivirals have been shown to shorten the duration of influenza symptoms (10). In observational studies, influenza antiviral medications have reduced the risk for severe complications (10). Antiviral treatment decisions should not be delayed while awaiting laboratory confirmation of influenza; rather, treatment should be administered as soon as possible for any patient with confirmed or suspected influenza at high risk for influenza-associated complications (10).

What is already known on this topic?

CDC collects, compiles, and analyzes data on influenza activity year-round in the United States. Substantial influenza activity generally begins in the fall and continues through the winter and spring months; however, the timing and severity of influenza activity varies by geographic location and season.

What is added by this report?

The 2014–15 influenza season was an influenza A (H3N2)–predominant and moderately severe season overall, but was especially severe for adults aged ≥65 years. This age group had the highest laboratory-confirmed influenza hospitalization rates and also accounted for the majority of pneumonia and influenza deaths. Antigenic and genetic characterization showed that most of the circulating influenza A (H3N2) viruses were different from the influenza A (H3N2) component of the 2014–15 Northern Hemisphere vaccines, resulting in reduced vaccine effectiveness.

What are the implications for public health practice?

Influenza vaccination remains the most effective way to prevent influenza illness and its associated complications. Although vaccine effectiveness was reduced this season because of antigenic drift in H3N2 viruses, vaccination was still protective against vaccine-like influenza A (H3N2) viruses and influenza B viruses. Timely influenza surveillance informs vaccine strain selection; the influenza A (H3) and influenza B components of the subsequent 2015–16 season vaccine have been changed to more optimally match circulating viruses. As an adjunct to vaccination, timely empiric antiviral treatment is also recommended for all patients with severe, complicated, or progressive influenza illness and those at higher risk for influenza-associated complications, including adults aged ≥65 years.

Influenza surveillance reports for the United States are posted online weekly and are available at http://www.cdc.gov/flu/weekly. Additional information regarding influenza viruses, influenza surveillance, influenza vaccine, influenza antiviral medications, and novel influenza A infections in humans is available at http://www.cdc.gov/flu.

## Footnotes

* The CDC influenza surveillance system collects information in five categories from eight data sources: 1) viral surveillance (World Health Organization collaborating laboratories, the National Respiratory and Enteric Virus Surveillance System, and novel influenza A virus case reporting); 2) outpatient illness surveillance (U.S. Outpatient Influenza-Like Illness Surveillance Network); 3) mortality (122 Cities Mortality Reporting System and influenza-associated pediatric mortality reports); 4) hospitalizations (Influenza Hospitalization Surveillance Network [FluSurv-NET], which includes the Emerging Infections Program and surveillance in three additional states); and 5) summary of the geographic spread of influenza (state and territorial epidemiologist reports).

† Data as of May 23, 2015.

§ Influenza viruses that normally circulate in pigs are called “variant” viruses when they are found in humans. Influenza A (H3N2) variant viruses (“H3N2v” viruses) with the matrix (M) gene from the 2009 H1N1 pandemic virus were first detected in humans in July 2011. Since then, 352 cases of H3N2v infection have been confirmed in humans, mostly associated with prolonged exposure to pigs at agricultural fairs. Of the other variant viruses, to date, 19 cases of H1N1v and five cases of H1N2v have been detected in humans.

¶ *Region 1:* Connecticut, Maine, Massachusetts, New Hampshire, Rhode Island, and Vermont. *Region 2:* New Jersey, New York, Puerto Rico, and the U.S. Virgin Islands. *Region 3:* Delaware, the District of Columbia, Maryland, Pennsylvania, Virginia, and West Virginia. *Region 4:* Alabama, Florida, Georgia, Kentucky, Mississippi, North Carolina, South Carolina, and Tennessee. *Region 5:* Illinois, Indiana, Michigan, Minnesota, Ohio, and Wisconsin. *Region 6:* Arkansas, Louisiana, New Mexico, Oklahoma, and Texas. *Region 7:* Iowa, Kansas, Missouri, and Nebraska. *Region 8:* Colorado, Montana, North Dakota, South Dakota, Utah, and Wyoming. *Region 9:* Arizona, California, Hawaii, Nevada, American Samoa, Commonwealth of the Northern Mariana Islands, Federated States of Micronesia, Guam, Marshall Islands, and Republic of Palau. *Region 10:* Alaska, Idaho, Oregon, and Washington.

** CDC routinely uses hemagglutination inhibition (HI) assays to antigenically characterize influenza viruses year-round to compare how similar currently circulating influenza viruses are to those included in the influenza vaccine, and to monitor for changes in circulating influenza viruses. However, a portion of recent influenza A (H3N2) viruses did not yield sufficient hemagglutination titers for antigenic characterization by HI. For many of these viruses, CDC performed genetic characterization to infer antigenic properties and is also using alternative methods (e.g., focus forming unit reduction) for antigenic characterization.

†† Defined as a temperature of ≥100.0°F (≥37.8°C), oral or equivalent, and cough or sore throat, in the absence of a known cause other than influenza.

§§ The national and regional baselines are the mean percentage of visits for ILI during weeks with little or no influenza virus circulation (non-influenza weeks) for the previous three seasons plus two standard deviations. A non-influenza week is defined as periods of ≥2 consecutive weeks in which each week accounted for <2% of the season’s total number of specimens that tested positive for influenza. National and regional percentages of patient visits for ILI are weighted on the basis of state population. Use of the national baseline for regional data is not appropriate.

¶¶ Activity levels are based on the percentage of outpatient visits in a jurisdiction attributed to ILI and are compared with the average percentage of ILI visits that occur during weeks with little or no influenza virus circulation. Activity levels range from minimal, which would correspond to ILI activity from outpatient clinics being at or below the average, to high, which would correspond to ILI activity from outpatient clinics being much higher than the average. Because the clinical definition of ILI is very nonspecific, not all ILI is caused by influenza; however, when combined with laboratory data, the information on ILI activity provides a clearer picture of influenza activity in the United States.

*** Levels of activity are 1) no activity; 2) sporadic: isolated laboratory-confirmed influenza case(s) or a laboratory-confirmed outbreak in one institution, with no increase in activity; 3) local: increased ILI, or at least two institutional outbreaks (ILI or laboratory-confirmed influenza) in one region of the state, with recent laboratory evidence of influenza in that region and virus activity no greater than sporadic in other regions; 4) regional: increased ILI activity or institutional outbreaks (ILI or laboratory-confirmed influenza) in at least two but less than half of the regions in the state with recent laboratory evidence of influenza in those regions; and 5) widespread: increased ILI activity or institutional outbreaks (ILI or laboratory-confirmed influenza) in at least half the regions in the state, with recent laboratory evidence of influenza in the state.

††† FluSurv-NET conducts population-based surveillance for laboratory-confirmed influenza-associated hospitalizations among children aged <18 years (since the 2003–04 influenza season) and adults aged ≥18 years (since the 2005–06 influenza season). FluSurv-NET covers approximately 70 counties in the 10 Emerging Infections Program states (California, Colorado, Connecticut, Georgia, Maryland, Minnesota, New Mexico, New York, Oregon, and Tennessee) and additional Influenza Hospitalization Surveillance Project (IHSP) states. IHSP began during the 2009–10 season to enhance surveillance during the 2009 H1N1 pandemic. IHSP sites included Iowa, Idaho, Michigan, Oklahoma, and South Dakota during the 2009–10 season; Idaho, Michigan, Ohio, Oklahoma, Rhode Island, and Utah during the 2010–11 season; Michigan, Ohio, Rhode Island, and Utah during the 2011–12 season; Iowa, Michigan, Ohio, Rhode Island, and Utah during the 2012–13 season; and Michigan, Ohio, and Utah during the 2013–14 and 2014–15 seasons.

§§§ Incidence rates are calculated using CDC’s National Center for Health Statistics population estimates for the counties included in the surveillance catchment area. Laboratory confirmation is dependent on clinician-ordered influenza testing, and testing for influenza often is underutilized because of the poor reliability of rapid influenza diagnostic test results and greater reliance on clinical diagnosis for influenza. As a consequence, the number of cases identified as part of influenza hospitalization surveillance likely is an underestimation of the actual number of persons hospitalized with influenza.

¶¶¶ The seasonal baseline proportion of P&I deaths is projected using a robust regression procedure, in which a periodic regression model is applied to the observed percentage of deaths from P&I that were reported by the 122 Cities Mortality Reporting System during the preceding 5 years. The epidemic threshold is set at 1.645 standard deviations above the seasonal baseline.
